# Alginate-Iron Speciation and Its Effect on *In Vitro* Cellular Iron Metabolism

**DOI:** 10.1371/journal.pone.0138240

**Published:** 2015-09-17

**Authors:** Richard D. Horniblow, Miriam Dowle, Tariq H. Iqbal, Gladys O. Latunde-Dada, Richard E. Palmer, Zoe Pikramenou, Chris Tselepis

**Affiliations:** 1 University of Birmingham, School of Cancer Sciences, College of Medical and Dental Sciences, Vincent Drive, Edgbaston, Birmingham, B15 2TT, United Kingdom; 2 University of Birmingham, School of Physics, College of Engineering and Physical Sciences, Edgbaston, Birmingham, B15 2TT, United Kingdom; 3 Diabetes and Nutritional Sciences, King's College London, Franklin Wilkins Building, 150 Stamford Street, London, SE1 9NH, United Kingdom; 4 University of Birmingham, School of Chemistry, College of Engineering and Physical Sciences, Edgbaston, Birmingham, B15 2TT, United Kingdom; Roswell Park Cancer Institute, UNITED STATES

## Abstract

Alginates are a class of biopolymers with known iron binding properties which are routinely used in the fabrication of iron-oxide nanoparticles. In addition, alginates have been implicated in influencing human iron absorption. However, the synthesis of iron oxide nanoparticles employs non-physiological pH conditions and whether nanoparticle formation *in vivo* is responsible for influencing cellular iron metabolism is unclear. Thus the aims of this study were to determine how alginate and iron interact at gastric-comparable pH conditions and how this influences iron metabolism. Employing a range of spectroscopic techniques under physiological conditions alginate-iron complexation was confirmed and, in conjunction with aberration corrected scanning transmission electron microscopy, nanoparticles were observed. The results infer a nucleation-type model of iron binding whereby alginate is templating the condensation of iron-hydroxide complexes to form iron oxide centred nanoparticles. The interaction of alginate and iron at a cellular level was found to decrease cellular iron acquisition by 37% (p < 0.05) and in combination with confocal microscopy the alginate inhibits cellular iron transport through extracellular iron chelation with the resulting complexes not internalised. These results infer alginate as being useful in the chelation of excess iron, especially in the context of inflammatory bowel disease and colorectal cancer where excess unabsorbed luminal iron is thought to be a driver of disease.

## Introduction

Alginates are a diverse class of biopolymers extracted from brown algae that are composed of 1–4 linked β-ᴅ-mannuronic acid (M) and α-L-guluronic acid (G) monomers. The polymers can vary in both chain length and composition which, in conjunction with their ability to interact with divalent metal cations, endows alginates with a wide range of physicochemical properties. Thus unsurprisingly alginates are widely used in the food industry, primarily due to their gelling capacity, and are also used in a range of medical applications, for example, in wound-healing preparations[1], controlled drug release systems[2,3] and anti-reflux formulations.[4] In addition, alginates are used for the construction of iron-oxide nanoparticles which have a myriad of applications from drug delivery to magnetic resonance imaging.[5]

The use of alginate as a scaffold for nanoparticle formulation is a well-accepted synthetic strategy, however, in these reactions the iron-oxide nanoparticles are fabricated using chemical-forcing conditions whereby highly basic conditions are used to form the iron hydroxide.[5–8] These conditions are considered optimal for the formation of iron oxide nanoparticles, with mean diameters ranging between 9 to 10 nm.[9,10] However, whether these iron oxide nanoparticles can form spontaneously in the gastrointestinal tract of man and in particular at low pH conditions of the stomach, (the first reasonable site of interaction between iron and alginate consumed in the diet) is unknown.

Supporting the concept of alginate binding iron, there is an increasing body of evidence emerging that alginates impact on iron metabolism in man.[11,12] A recent human study identified that an alginate supplemented diet resulted in decreased serum iron levels, alluding to the role of alginates chelating iron and thus limiting its absorption in the small bowel.[12] On the contrary, cellular studies have previously shown alginate as having an enhancing effect on intracellular iron concentration as assessed by ferritin expression; a surrogate biomarker for cellular iron levels.[13] However, whether these observed changes in cellular iron metabolism are related to the formation of alginate-iron nanoparticles is unknown. Interestingly, iron-oxide nanoparticles have been studied with respect to their cellular uptake with a potential application in iron fortification to treat anaemia.[14–16] No toxicity was associated with the uptake of these nanoparticles and results indicated that these Fe(III) nanoparticles were directly taken up by enterocytes *in vitro* and markedly increased cellular iron concentrations.

Thus, the existing published literature is inconsistent and it remains unclear how alginates might interact with iron in the context of the gut and whether any resulting complexation may be of use as a platform for iron fortification or chelation.

Therefore, the aims of this study are i) to determine how alginate and iron(III) interact at gastric-comparable pH conditions; ii) to verify the speciation of iron with alginate upon complexation under these conditions and iii) to understand how alginate modulates cellular iron status.

## Materials and Methods

### Alginate preparation

Sodium alginate LFR5/60 was a kind gift of FMC Biopolymer, Norway. The average molecular weight is 34700 Da and a G/M composition of 65%/35%. Dispersion of alginates into water was achieved through high vortex stirring and solutions left overnight to ensure full hydration.

### Isothermal Titration Calorimetry

An adapted protocol was used whereby iron was titrated into LFR5/60 at 37°C.[17] Isothermal Titration Calorimetry (ITC) measurements were performed on a VPITC MicroCalorimeter and data was automatically analysed using MicoCal LLC ITC/Origin software package; the binding isotherm was obtained by integrating injections and fitting them to an appropriate binding model. All alginate solutions were excessively dialysed before use, to eliminate errors caused by pH and ionic strength mismatches, and also degassed before use at 2°C below the titration temperature. Typical experimental parameters included: 37°C cell temperature, 10 μcal s^-1^ reference power, stirring speed at 286 rpm with the initial injection being small (2 μl) and discarded in the data analysis. Titration was performed by injection of 8 μl aliquots of aqueous iron (III) chloride (5mM) in DI H_2_O into a solution of aqueous LFR5/60 (0.07 mM, pH = 5.8) in DI H_2_O. A delay of 350 sec between each injection was set to allow the energy difference to return back to baseline. To account for the high energy changes associated with iron titration into water (the control titration), these heat integrations were subtracted from that of the alginate-iron titration and the subsequent heats of interaction were fitted using a model of two binding sites.

### Equilibrium dialysis preparation of alginate-iron composites

Alginate solutions (0.1% w/v, 10 ml) were sealed into a dialysis membrane (M_r_ cut off = 12,400 Da) and incubated in aqueous FeCl_3_∙6H_2_O (10 mM, 750 ml) for 120 min and washed in deionised water for a subsequent 120 min. The pH changes of the alginate were tracked over the period of the incubation. The pH of the alginate inside the dialysis bag pre-incubation was 5.8 and the pH of iron solution pre incubation was 1.7, equivalent to gastric acidity.[18] The pH of the alginate after incubation was 1.9 and after the wash period this was 3.7.

### UV-visible spectroscopy

Absorption spectra were recorded on a Varian Cary50 spectrometer using a quartz cuvette, path length 1 cm. Increments of varying volumes of aqueous FeCl_3_∙6H_2_O (10mM) in DI H_2_O were titrated into a stirred solution of aqueous alginate (2 mL, 0.1% w/v) in DI H_2_O, allowed to equilibrate for several seconds and then scanned. Measurements were taken up to a point of saturation. To correct for the absorption of aqueous-iron species at the wavelengths of interest difference absorbance spectra were obtained by correction of the alginate titration with the equimolar iron-water control titration.

### CD-spectroscopy

CD measurements were recorded on a Jasco J-810 CD spectropolarimeter (4 accumulations with 1 s response) using a 1cm path length, blackened quartz cell. Samples for CD measurements were prepared as described for the dialysis preparations above.

### Cell culture

Human RKO colorectal carcinoma cells (obtained from the ACTT CRL-2577) were routinely cultured in Dulbecco’s modified eagles medium including 10% v/v foetal calf serum, 100 units/ml penicillin and 0.1 mg/ml streptomycin. Cells were seeded in six well plates at a concentration of 1x10^5^ cells/ml and grown in medium alone for 24 hours. Once established, the growth medium was removed and supplemented medium was added (Iron loaded medium: 100 μM FeSO_4_∙7H_2_O and 10 μM sodium ascorbate or alginate loaded medium: LFR5/60 (0.3% (w/v)), 100 μM FeSO_4_∙7H_2_O and 10 μM sodium ascorbate)) and incubated for 24 hours. Preparation of these media was performed as follows; aqueous FeSO_4_∙7H_2_O (100 μL, 10 mM) containing sodium ascorbate (500 mM) in DI H_2_O was added to a sample of aqueous sodium alginate (1.5 mL, 2% w/v) in DI H_2_O and mixed. Growth medium was then added (8.4 mL) and all constituents were thoroughly mixed. Where alginate was not supplemented, growth medium (9.9 mL) was added to the iron only. After 24 hours the medium was removed and the cells washed three times with phosphate buffered saline (PBS). Cells were lysed on ice in RIPA buffer (nonyl phenoxypolyethoxylethanol 1% (v/v), sodium deoxycholate 0.5% (w/v) and sodium dodecyl sulphate 0.1% (w/v)) containing protease inhibitors. All samples were then sonicated for 10 sec whilst kept at 4⁰C. A protein assay kit (Peirce BCA protein assay) was used to determine the protein concentration in each sample.

### Western blotting

Western blotting was performed as previously described, with monoclonal antibodies to ferritin (1:5000, Abcam, Rabbit AB69090) and β-actin (1:5000, Abcam, Mouse AB8226).[19] All blots were subject to densitometry analysis using ImageJ analysing software and data normalised to respective β-actin loading controls.

### Synthesis of FITC-alginate

Synthesis of FITC-alginate was performed according to the protocol of Strand *et al*. [20] Sodium alginate LFR5/60 (0.32 g, 9.2X10^-6^ moles) was dissolved in a solution of phosphate buffered saline at pH 7.4 (50 ml). 1-ethyl-3-(3-dimethylaminopropyl)carbodiimide) (0.018 g, 9.2X10^-5^ moles) and n-Hydroxysulfosuccinimide sodium salt (0.02 g, 9.2X10^-5^ moles) was added to the alginate solution and stirred for two hours. Once mixed, fluoresceinamine (0.0319 g, 9.2X10^-5^ moles) was added and the reaction left to stir in darkness for 24 hours. Free, unreacted fluorophore was removed by extensive dialysis; one wash in deionised H_2_O (3.5 L) at 4⁰C for 24 hours, three washes in NaCl (3.5 L, 1 M) for 24 hours each, then a subsequent six washes in deionised H_2_O (3.5 L) for 24 hours each. Once purified, the pH of the solution was adjusted to 7.4 and stored in the dark at 4⁰C until required.

### Confocal microscopy

Slides for confocal microscopy were prepared by growing RKO cells on sterile 22 mm cover slips placed in individual wells of a six well cell culture plate. Cells were seeded in six well plates at a concentration of 1X10^5^ cells/ml and grown in medium alone for 24 hours. Once established, cell permeabilisation was performed using saponin (50 μg/ ml) as previously described.[21] The growth medium was replaced with fluorescent-alginate loaded medium (0.04% fluorescent alginate, 100 μM FeSO_4_∙7H_2_O and 10 μM sodium ascorbate) and incubated for 24 hours. Cell nuclei and plasma membranes were subsequently stained with Hoechst 33450 (NucBlue Live Cell Stain, Life technologies) and CellMask Deep Red plasma stain, (Life technologies) respectively and cells fixed with a 4% paraformaldehyde solution (pH 7.4) at room temperature. Once fixed images were captured using a Zeiss LSM510 META confocal system with x63 1.4 oil immersion objective.

### Intracellular iron assessment

Cells were seeded in six well plates at a concentration of 1X10^5^ cells/ml and grown in medium alone for 24 hours. Once established, the growth medium was removed and supplemented medium was added (Iron loaded medium: 100 μM FeSO_4_∙7H_2_O and 500 μM sodium ascorbate, or alginate loaded medium: LFR5/60 (0.3% (w/v)), 100 μM FeSO_4_∙7H_2_O and 500 μM sodium ascorbate)) and incubated for 24 hours; in both instances iron stimulations were spiked with ^59^FeCl_3_ to reach ca. 10,000 counts per minute (CPM) per well. To prepare the radio-active iron medium with alginate, aqueous FeSO_4_∙7H_2_O (100 μL, 10 mM) in DI H_2_O containing sodium ascorbate (500 mM) in DI H_2_O was mixed with ^59^FeCl_3_ in aqueous HCl (0.1M). This was then added to aqueous sodium alginate (1.5 mL, 2% w/v) in DI H_2_O and thoroughly mixed. Growth medium was then added (8.4 mL) and all constituents mixed. In iron-only supplemented media, no sodium alginate was added, only the addition of growth medium (9.9 mL) to the radio-active iron. After this incubation period the medium was removed and cells washed three times with 2 ml Versene (0.2g/L EDTA in phosphate buffered saline), and lysed in 150 μl HEPES-saline lysis buffer (10 mM, pH 7.4, NaCl 0.9% (w/v)). To determine cellular iron content, the lysates were pipetted into scintillation tubes containing scintillation fluid (1 mL, PerkinElmer OPTIPHASE HISAFE3) and counted on a gamma-counter (Packard 2500 TR liquid scintillation counter).

### Scanning Transmission Electron Microscopy/ Energy-dispersive X-ray spectroscopy

Alginate-iron samples used for STEM/EDX were made using the equilibrium dialysis technique as described earlier. Preparation of these samples was performed as follows; aqueous sodium alginate (10 mL, 0.1% w/v, pH 5.8) in DI H_2_O was sealed within a dialysis membrane and immersed in a pre-mixed solution of FeCl_3_∙6H_2_O (10 mM, 750 ml, pH 1.7) for 120 min. The dialysis bag was removed, and subsequently immersed in pure DI H_2_O (750 mL) and incubated for another 120 min. Due to the viscous nature of the sample, copper TEM grids coated with lacey carbon were loaded with 50 μl of sample and excess sample was drawn from underneath, effectively pulling the sample through the grid. This produced a thin sample coverage over the grid with many sampling areas.

Electron microscopy images were taken using a 200kV FEG Jeol 2100F scanning transmission electron microscope fitted with a CEOS aberration corrector. Images were simultaneously acquired in high angular annular dark field (HAADF) and bright field (BF) mode using the Gatan DigitalMicrograph software package.

## Results

### Studies of iron-alginate complexation

**Fig 1:**
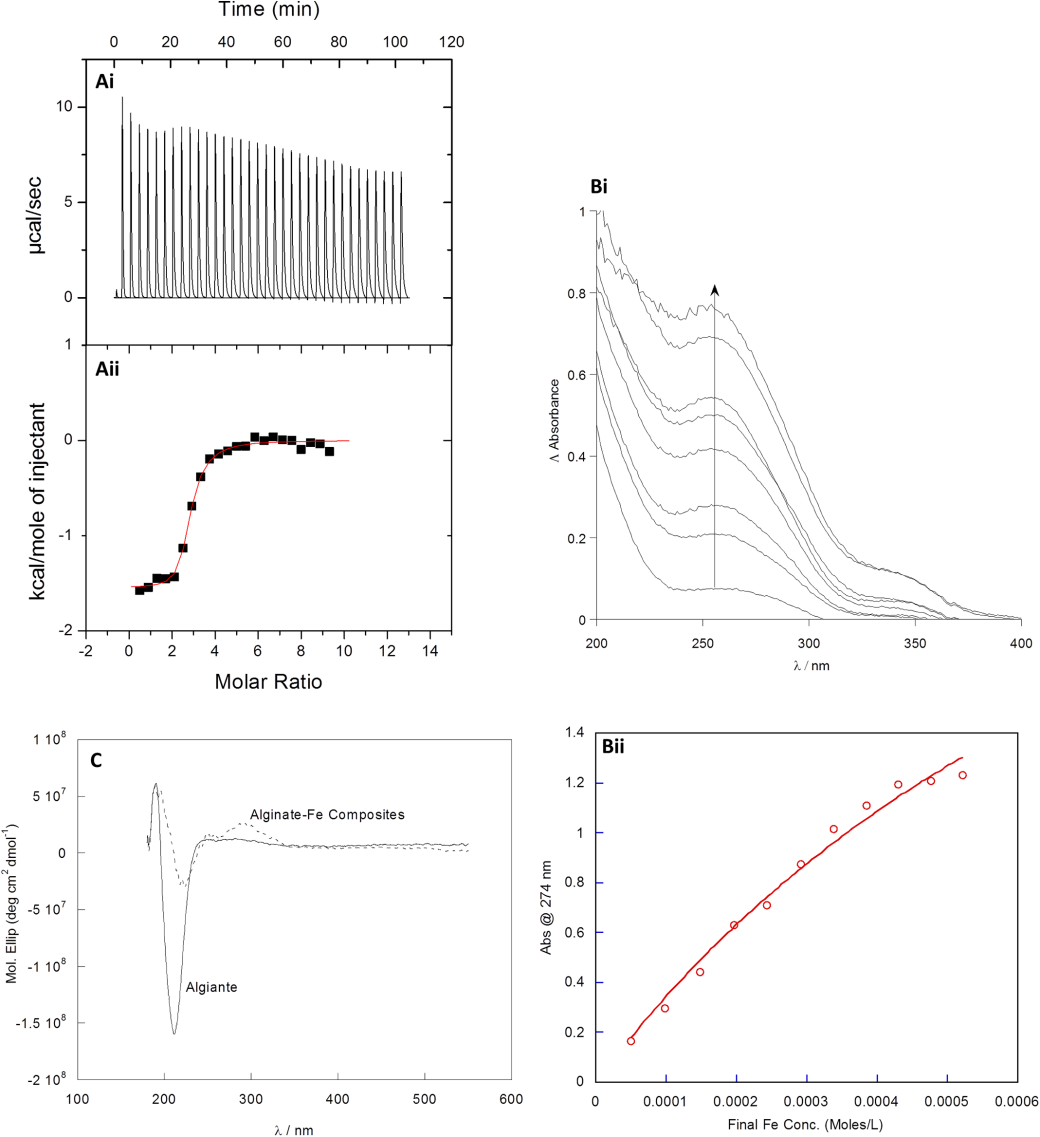
Chemical analysis of iron alginate binding. (Ai) Isothermal titration microcalorimetry thermogram of 8 μl injectants of 5 mM Fe(III) into 0.04 mM alginate at 37°C. (Aii) Corresponding isotherm. (Bi) UV-Visible difference spectra of iron (III) titrated into alginate with a clear absorbance change at ca. 280 nm (Bii) absorbance change at 274nm vs final Fe concentration (M) with binding curve) (C) CD spectra of alginate-iron composites isolated via equilibrium dialysis. An induced CD signal is evident at ca. 280 nm. This correlates to the iron-hydroxide species bonded to the alginate as indicated from the UV-Visible spectra.

Isothermal titration microcalorimetry (ITC) was performed to examine the strength of interaction between iron and alginate (LFR5/60). Iron(III) chloride was titrated into a solution of LFR5/60 and a drop in the integrated heats of each addition was observed (Fig 1A) Saturation of iron binding by alginate occurred at a molar ratio of iron:alginate of 3:1, with a 5 times excess of iron used to ensure saturation of all the binding sites on the alginate. Data analysis, which was best fit using a two-site binding model, revealed two binding events between alginate and iron with the estimated binding constants calculated as K_1_ = 1X10^6^ and K_2_ = 3X10^4^ M^-1^ respectively. The equation that models this binding can be found in S1 Fig. Entropy was positive in both binding events (18.4 and 23.2 cal mol^-1^ K^-1^). Enthalpy values were found to be exothermic for both binding events (-704 x 10^4^ and -1548 cal mol^-1^).

The binding of ferric ions to alginate was further verified using UV-Visible and Circular Dichroism (CD) spectroscopy. (Fig 1B and 1C) Titration of an aqueous solution of Fe(III) to an aqueous solution of sodium alginate LFR5/60 revealed the growth of a band at 280 nm (Fig 1C); confirming iron binding to alginate. Profile changes were plotted against molar equivalents of iron and a binding plot for alginate iron binding was obtained (Fig 1B). This data can be fitted to a 1:1 binding equation and an alginate iron binding constant of K = 1X10^3^ M^-1^ calculated (S2 Fig).

To further support the spectroscopic results demonstrating alginate complexation, CD spectroscopy was also performed since its fundamental application is probing transitions within chiral compounds; alginate is highly chiral due to its polymeric nature and the chiral carbon centres on the individual monomers. Alginate-iron complexes were isolated using equilibrium dialysis. The CD spectrum of the isolated iron-alginate complexes shows the appearance of a peak at 280 nm (Fig 1C), which correlates with the peak identified in the UV-visible spectrum (Fig 1B). This profile is indicative of iron hydroxide (Fe-OH) binding and confirms that the changes observed in the UV-visible spectrum are attributed to the alginate binding to Fe-OH species in solution (Fig 1C).

### Alginates template iron oxide nanoparticle formation under simple mixing conditions

**Fig 2:**
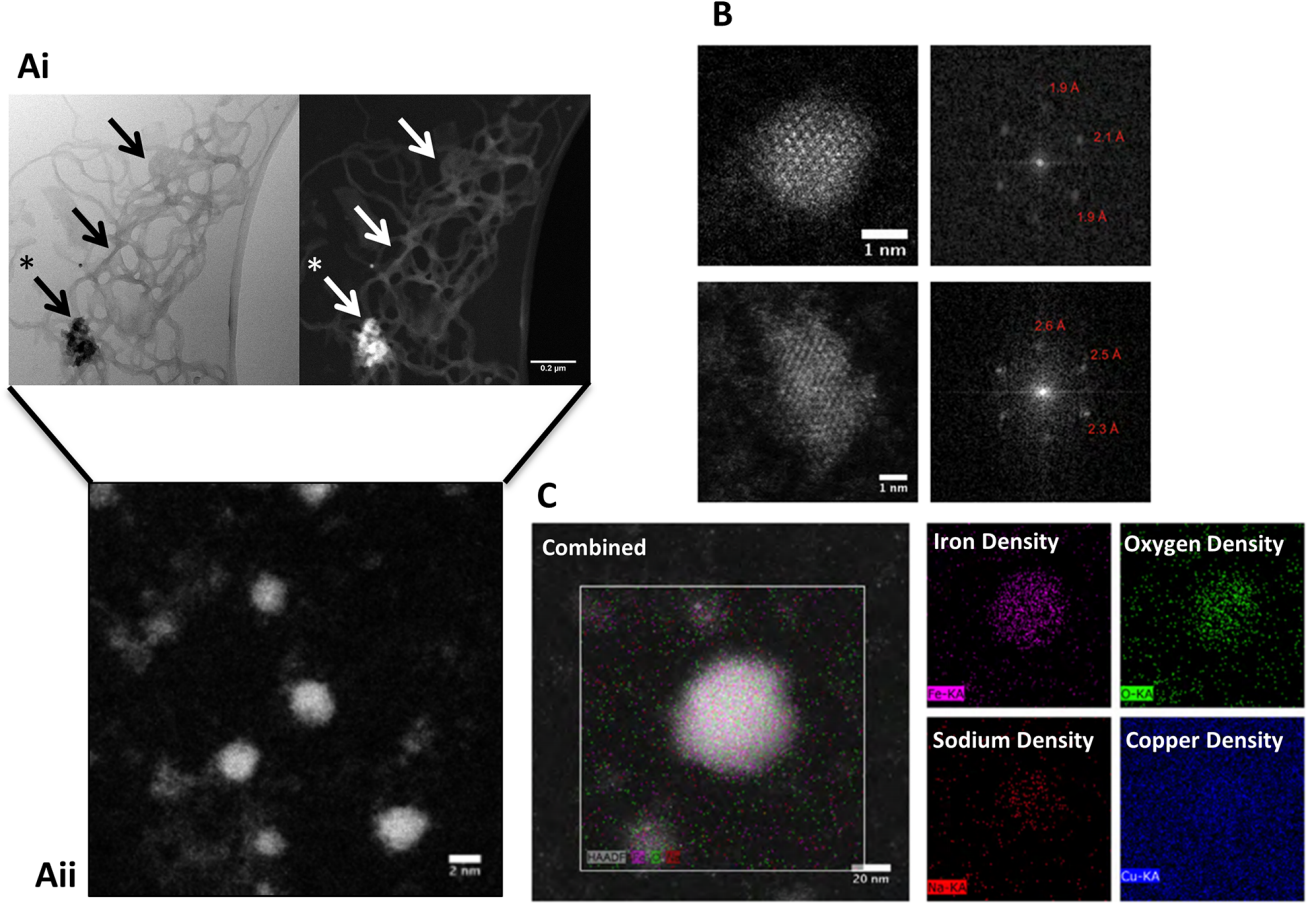
Physical characterisation of alginate iron composites. (Ai) Low magnification STEM images of alginate-iron composites revealed the alginate network ‘decorated’ in iron (denoted by arrows) with a single highly dense iron nucleation site (denoted with an asterisk). (Aii) A higher magnification image of the nucleation centre revealed nanoparticles of approximately 2–5 nm in diameter. (B) Fast Fourier transform analysis of HAADF-STEM images of two individual nanoparticles. (C) EDX mapping of iron-alginate composites with oxygen, iron and sodium localisation shown in the sample area. The copper from the copper TEM grid functions as a control.

As CD identified the presence of Fe-OH within the alginate-iron composites, these were further characterised using aberration corrected STEM (Fig 2). This is capable of achieving ultra-high resolution with atomic number contrast imaging in the material sciences, but is rarely used to investigate ‘biological’ samples due to the low atomic number of most bio-materials.[22] However, the iron component of the alginate-iron composites provided distinguishable contrast, thus it was possible to image atomic structure at the highest resolution (Fig 2).

Low magnification HAADF-STEM imaging revealed a gel-like alginate network covered in iron with dense nucleation-sites present (Fig 2A) while energy-dispersive X-ray spectroscopy confirmed that these dense centres indeed contained iron (Fig 2C). Within this gel, small nanoparticles with a mean diameter of 1.78 ± 0.70 nm were detected and under higher magnification lattice structures could be visualised within these nanoparticles (Fig 2B). Fast Fourier transform analysis of the lattice arrangements gave diffraction spots (labelled) which were in partial agreement with both Fe_2_O_3_ hematite_,_ and ferrihydrite, but the small particles yielded insufficient visible bright spots to determine the precise phase of these nanoparticles.[6,23] Samples of aqueous iron chloride alone were imaged and there was no evidence of particulate iron.

### The effect of alginate on cellular iron metabolism

**Fig 3:**
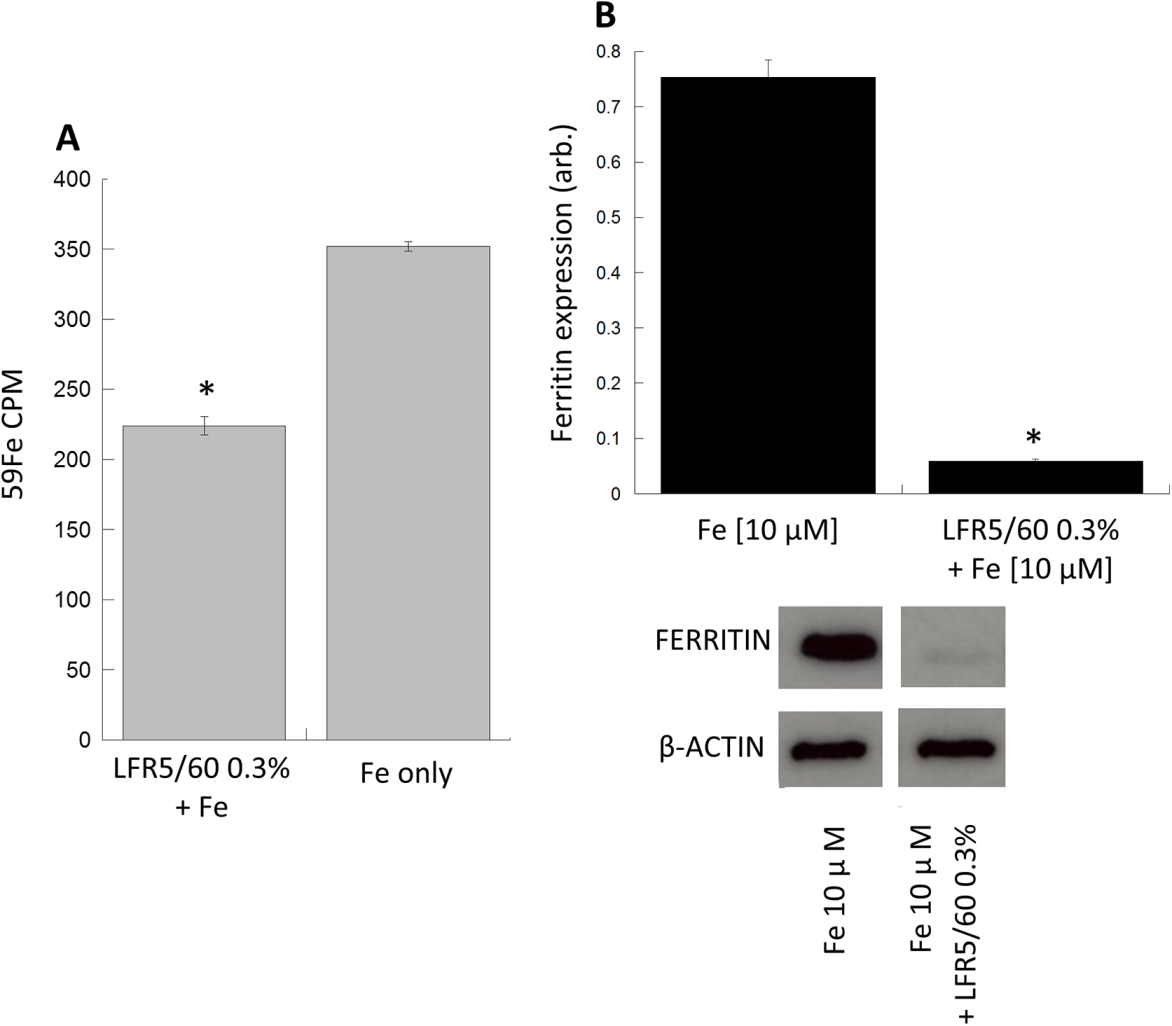
Effects of alginate on cellular iron transport. (A) Intracellular iron concentration decreases when RKO cells were incubated with iron-59 and alginate (0.3% w/v) compared to iron only control (B) Treatment of RKO cells with iron increases ferritin expression whilst co-incubation with alginate (0.3% w/v) significantly suppressed the iron mediated ferritin induction. All experiments were performed in triplicate with error bars representing +/- SEM and * denotes statistical significance at p < 0.05.

To assess the influence of alginate-iron interaction in human intestinal cells, RKO cells were challenged with an iron-enriched media either with, or without alginate LFR5/60. The iron-enriched media was spiked with radio-active iron-59, and after an incubation period of 24 hours the iron-content of the cell was measured (Fig 3A). Results demonstrated a significant decrease (37%, p < 0.05) in radioactivity in cells co-cultured in the presence of alginate compared to control (no alginate). To support this result ferritin expression, a surrogate marker for cellular iron levels was also determined. Ferritin expression was significantly decreased (17 fold; p < 0.05) when cells were cultured in the presence of iron and alginate compared to iron alone (control) (Fig 3B).

### Alginate iron composites are not cell-permeable

**Fig 4:**
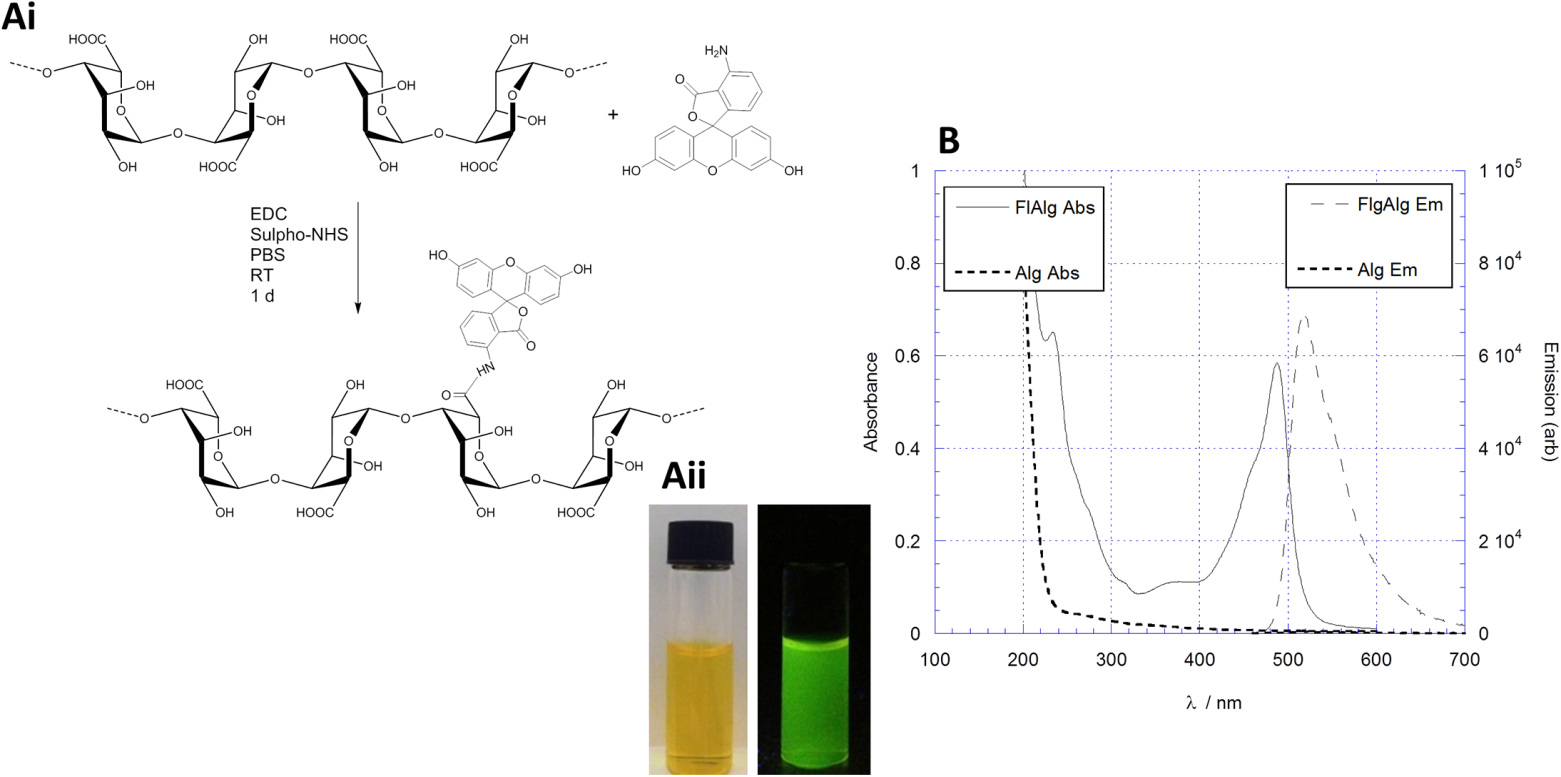
Synthesis of FITC alginate. (Ai) Reaction coupling scheme of FITC onto alginate under peptide coupling conditions. (Aii) Image of fluorescent alginate in normal light (left) and exposed to λ = 365 nm UV light (right). (B) Absorption and emission (red and blue lines respectively) spectra of the fluorescent alginate (FlAlg) product. The native alginate reactant has no absorption or emission profile, however, upon conjugation with FITC a highly absorption and emission peaks are observed.

Since alginate depleted intracellular iron, confocal microscopy was performed to assess the localisation of the alginate in these cell culture experiments and specifically to deduce if the alginate is internalised. To assess the bioavailability of alginate LFR5/60 and the composites formed upon interaction with iron a fluorescent analogue was prepared by conjugation with fluoresceinamine (FITC) as schematically illustrated in Fig 4Ai. Absorption and emission spectra were recorded for the fluorescent alginate with absorption and emission maxima (λ_max_) at ca. 490 and 550 nm respectively (Fig 4B).

**Fig 5:**
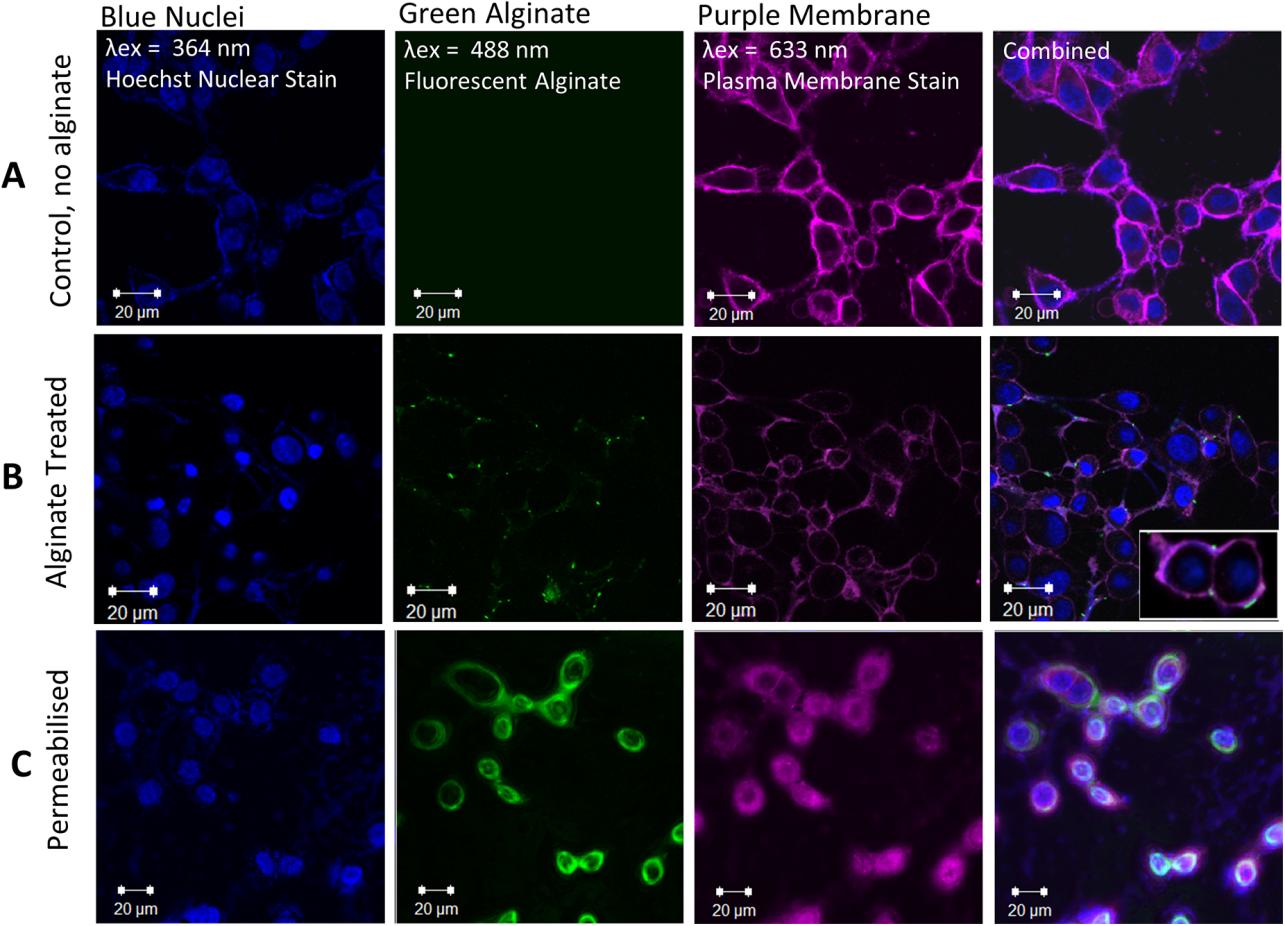
Cellular localisation of alginate with confocal microscopy. Cells were treated with iron alone (control) or iron and FITC alginate with or without cell-membrane permeabilisation. (A) Cells treated with iron alone as expected showed no FITC signal. (B) Cells treated with iron and FITC alginate showed negligible punctate FITC staining on the cell periphery (C) Cells permeabilised with Saponin and then cultured with iron and FITC alginate showed an abundance of intracellular FITC signal which was mostly cytoplasmic in localisation.

RKO cells were then cultured in the presence of FITC-alginate with or without a cellular permeabilisation step for 24 hours on microscope slips which were subsequently used for imaging in confocal microscopy. Cells were stained with DeepRed and Hoechst to define the cellular membrane and nucleus respectively (Fig 5).

Confocal image analysis revealed that whilst there was negligible amounts of FITC-alginate bound on the cell periphery (Fig 5B) no FITC-alginate was observed within the cell (Fig 5). As a further control cells were membrane-permeabilised and co-cultured with FITC-alginate, and in this instance, FITC- alginate is able to penetrate the outer cell membrane supporting the limited bioavailability of alginate in non-permeabilised cells.

## Discussion

Alginates and their use in nanoparticle formation is well established, however this is the first study to examine the interaction of iron and alginate in gastric-comparable pH conditions. This is particularly warranted since the existing literature is inconsistent in terms of the effect of alginate on cellular iron absorption and ultimate effects on human iron metabolism.[12,13]

Our results unequivocally demonstrate that alginate chelates iron under gastric comparable conditions as evidenced through UV Visible spectroscopy, ITC and CD. We have found by ITC that alginate–iron complexation involves two distinct binding events; an initial iron binding which then facilitates alginate reorganisation to accommodate the final iron binding. This is consistent with previous studies which indicate cooperativity of metal binding to alginate. In addition structural reorganisation of alginate polymers during ion binding has been reported previously in the case of calcium. [24,25]

With regards to the species of iron that is complexed to alginate, UV Visible spectroscopy, upon iron titration, revealed a peak at 280 nm which has previously been attributed to the presence of an iron-oxide species. This electronic transition is characteristic to the charge transfer originating from the OH- ligands to the Fe ion.[26] CD Spectroscopy demonstrated that the Fe-OH species were complexed to alginate since an induced CD signal was observed at 280 nm.

To probe the physical structure of the alginate iron composites formed under these physiological conditions HAADF-STEM was utilised. Interestingly, we demonstrate that alginate chelates iron to form a range of composites, from long range gel-like structured strands to smaller nanoparticulate matter. This mix of composites is unsurprising since the way the alginate and iron is brought together is uncontrolled. The combination of solution spectroscopy and high resolution STEM identified the core of the alginate nanoparticles to be iron-oxide in composition. However, it is known that the electron beam energy can affect samples under investigation during examination in an electron microscope and thus it is important to note that there is the possibility of electron beam damage causing structural and/or chemical particle-phase conversion. This could indeed result in a phase conversion from, for example, a ferrihydrite-particle to a haematite arrangement, which were both found here.[27]

**Fig 6:**
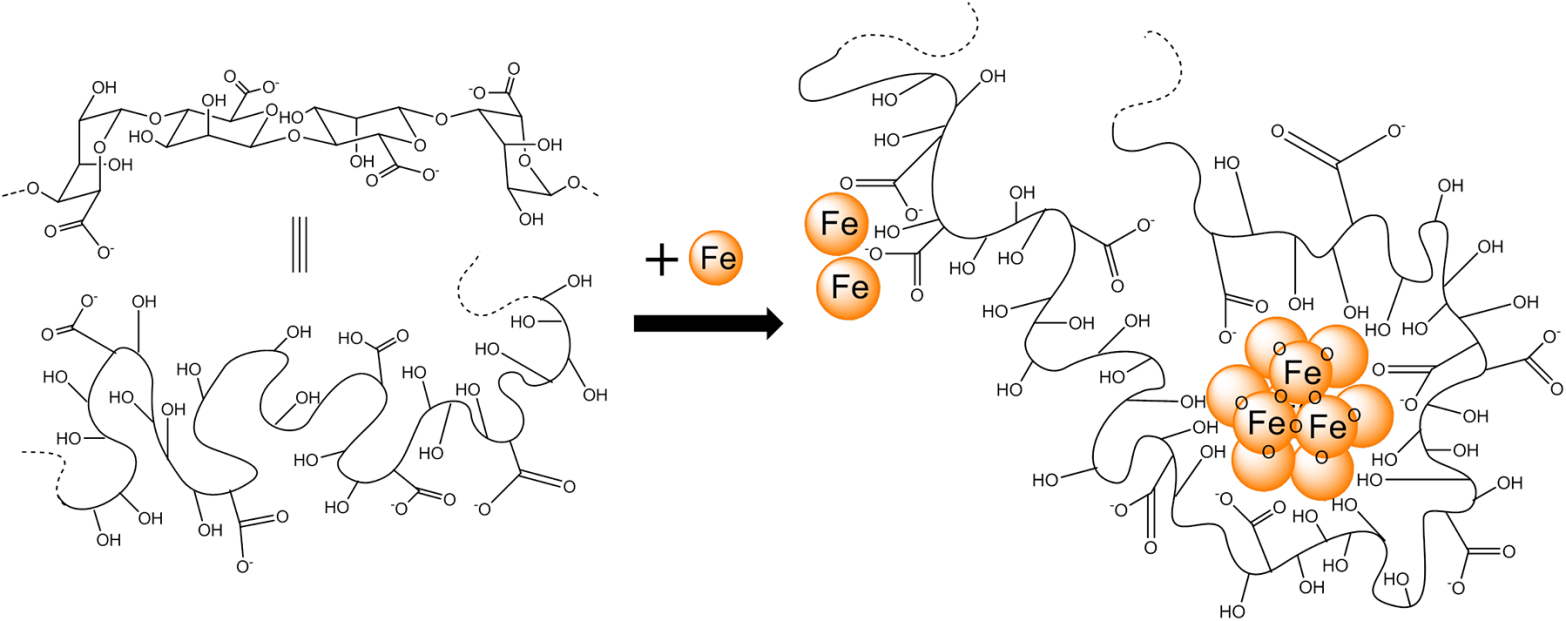
Schematic illustration of the binding arrangement of iron to alginate under simple mixing conditions.

A mechanism of nanoparticle formation can be proposed whereby iron initially binds to alginate forming an iron-hydroxide. Subsequently, at a critical concentration of iron loading, the alginate collapses and forms a nucleation site where iron hydroxide condensation can take place to form iron-oxide centred nanoparticles; the whole process templated by alginate (Fig 6). This mechanism supports the two event binding observed by ITC. Such models have been reported for other biopolymers binding metals including carrageenans. [28]

These findings confirmed the iron-binding ability of alginate, with iron binding constants on par with other biological proteins (e.g. ferritin), [29] and interestingly, ferritin similarly stores iron as its oxide form.[29,30] The formation of these composites may rationalise some of the effects seen in biological systems by others [12]. However, whilst it is clear that the alginate binds iron, what effect this has on cellular and human iron metabolism remains controversial whilst some studies advocating the usefulness of alginate in iron fortification and others iron chelation programmes. [13,31,32]. Our data suggest that alginate has the potential to bind ‘free’ reactive iron and this leads to the formation of iron-oxide centred nanoparticles. Although whether such ‘free’ iron exists within the gastrointestinal tract and or what form the residual unabsorbed iron takes still remains to be elucidated; indeed, there is evidence supporting the presence of both the particulate and ‘free’ forms.[33] Whether alginate is able bind particulate iron, if it exists within the gastrointestinal tract is not known and this clearly warrants further study.

From our own studies it is clear that alginate inhibits cellular iron transport by binding the iron in solution and the resulting complex remaining extracellular. This is evidenced by a suppression in cellular iron transport in the presence of alginate and a lack of any notable alginate present within the cells.[11,34–36] We may hypothesise from these observations that the alginate-iron nanoparticles are unable to be internalised into cells which may be due to a number of factors including nanoparticle size and surface coating.

Thus it can be considered that alginates act as iron chelators and are not bioavailable with respect to cellular uptake. This confirms previous studies, most notably, a recent human study has shown that alginate supplementation inhibits iron absorption in man, [11,36] and this may be attributed to the formation of iron-alginate nanoparticles. Thus *in vivo* alginate could be considered as an iron chelator and coupled with its inherent non-absorbability would make it an ideal candidate for use in patients with inflammatory bowel disease and colorectal cancer where excess reactive luminal iron is thought to be involved in the disease process.[37–39] One might predict that alginate supplementation in these groups would enhance their health through iron chelation.

## Supporting Information

S1 Fig ITC model of independent binding sites. Where Q = heat content of the solution, n = number of binding sites, M_t_ = total concentration of macromolecule in V_o_, V_o_ = active cell volume, H = enthalpy, X_t_ = total ligand concentration and K = the binding constant.
(TIF)

S2 Fig Equation of the 1:1 binding model. Where [H] = [alginate] and [G] = [Fe].
(TIF)
