# Supplementary figures and images for: Alginate-Iron Speciation and Its Effect on In Vitro Cellular Iron Metabolism
Source: PLoS One. 2015 Sep 17;10(9):e0138240. doi: 10.1371/journal.pone.0138240 (PMC4574481; doi:10.1371/journal.pone.0138240)

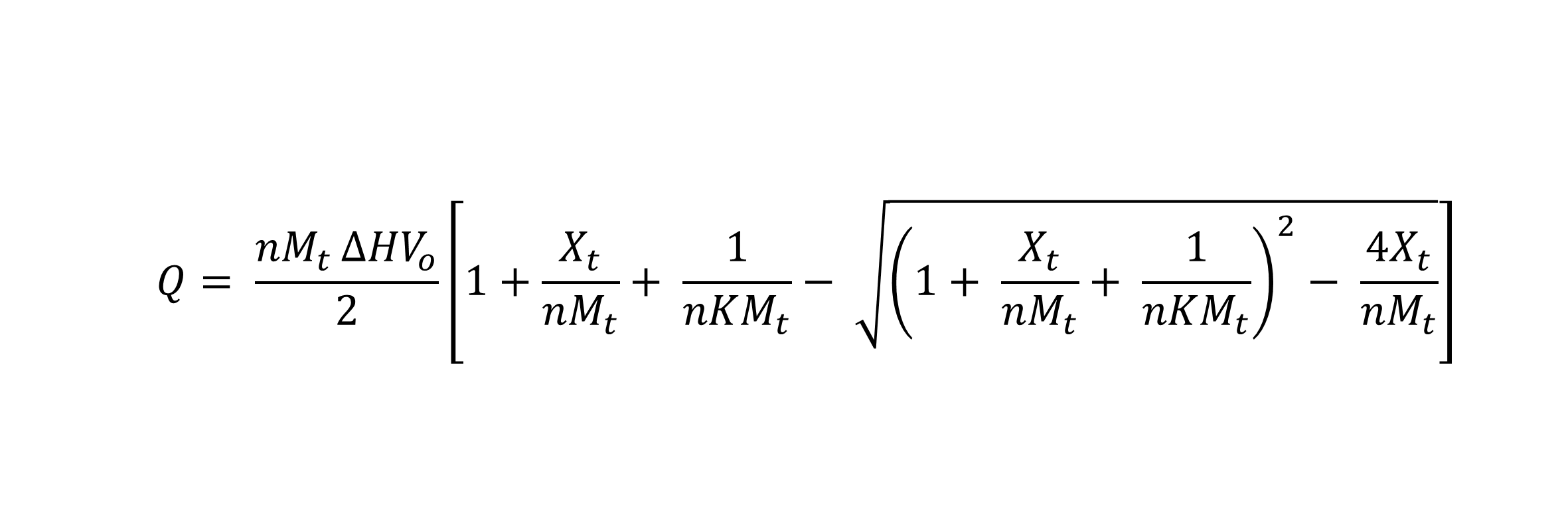

Supplement: S1 Fig — Where Q = heat content of the solution, n = number of binding sites, Mt = total concentration of macromolecule in Vo, Vo = active cell volume, H = enthalpy, Xt = total ligand concentration and K = the binding constant. (TIF) [file pone.0138240.s001.tif]

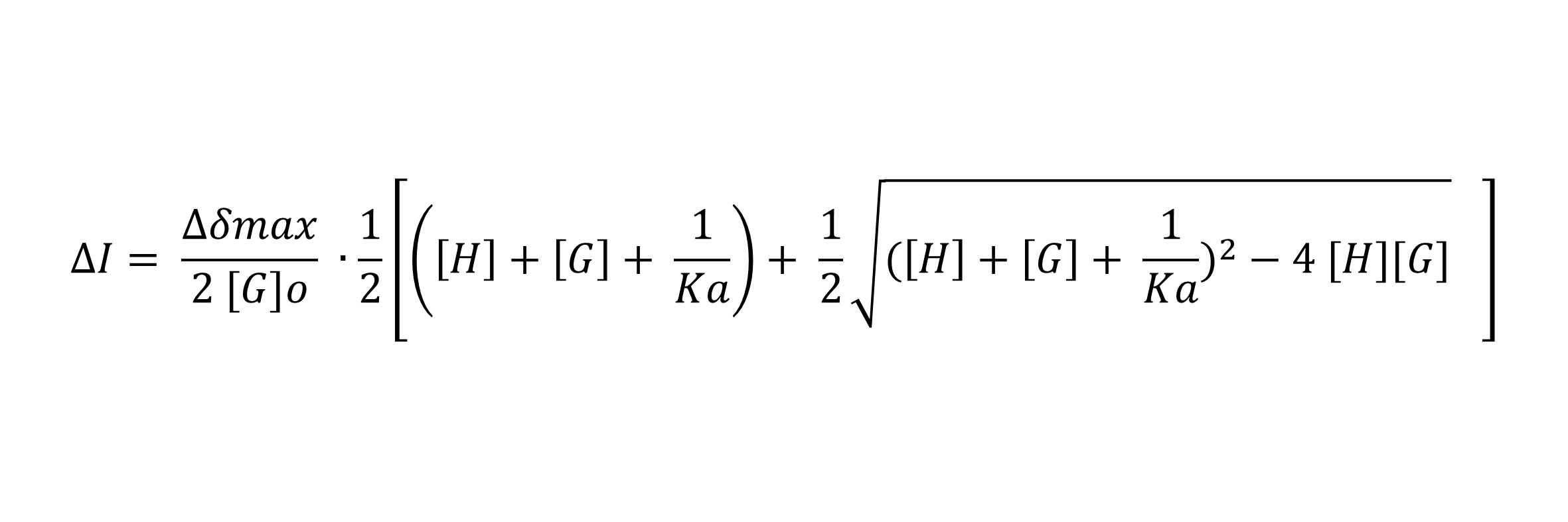

Supplement: S2 Fig — Where [H] = [alginate] and [G] = [Fe]. (TIF) [file pone.0138240.s002.tif]
